# Supplementary material for: Waterlogging Priming Enhances Hypoxia Stress Tolerance of Wheat Offspring Plants by Regulating Root Phenotypic and Physiological Adaption
Source: Plants (Basel). 2022 Jul 28;11(15):1969. doi: 10.3390/plants11151969 (PMC9370225; doi:10.3390/plants11151969)
Supplement: Supplementary file 1 [file plants-11-01969-s001.zip › plants-1742346-supplementary.pdf]

Supplementary figure

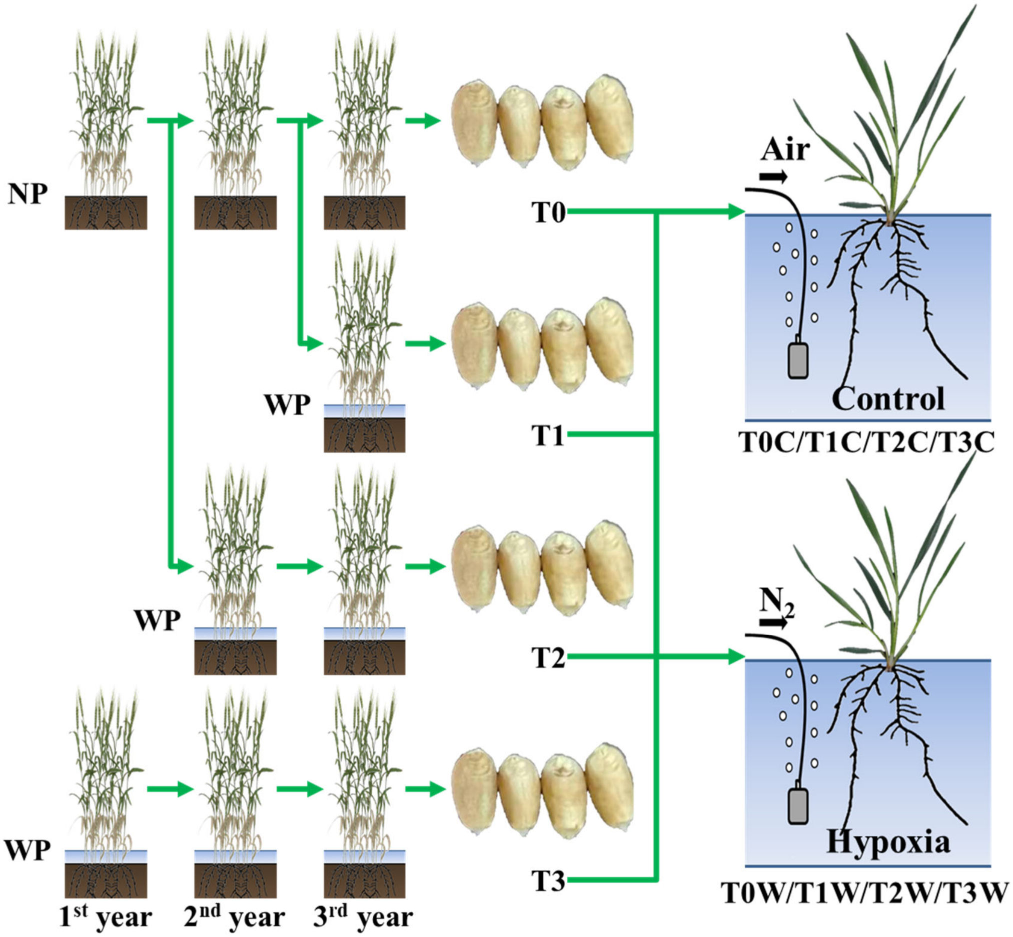

Figure S1. Waterlogging-priming pattern

Note: T0C, no-priming + no offspring hypoxia stress; T0W, no priming + offspring hypoxia stress; T1C, one-generation priming + no offspring hypoxia stress; T1W, one-generation priming + offspring hypoxia stress; T2C, two-generation priming + no offspring hypoxia stress; T2W, two-generation priming + offspring hypoxia stress; T3C, three-generation priming + no offspring hypoxia stress; T3W, three-generation priming + offspring hypoxia stress.

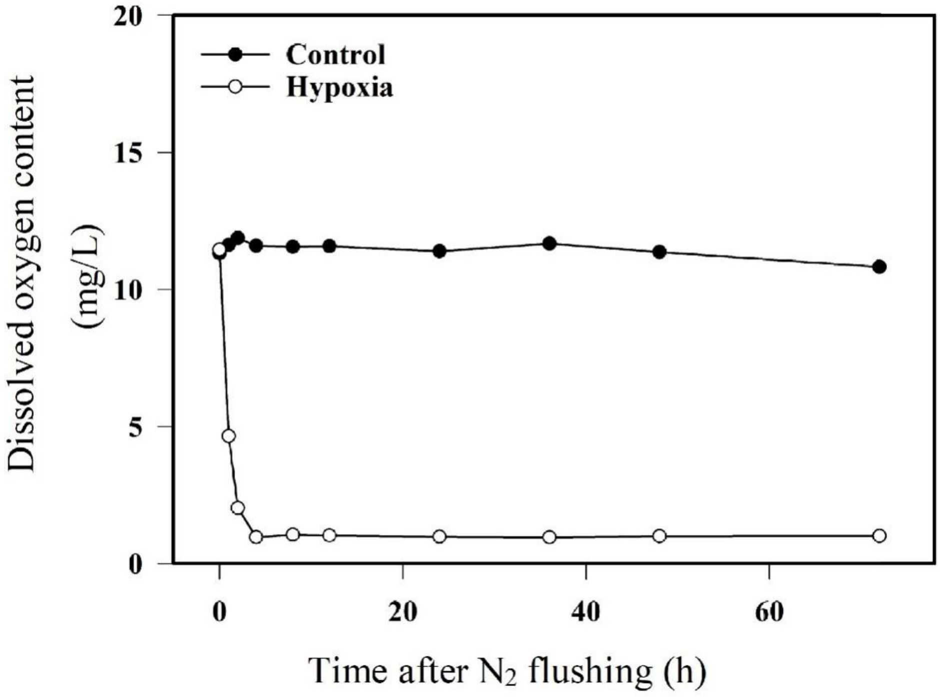

Figure S2. Dissolved oxygen content changes over time in solution with aeration of nitrogen

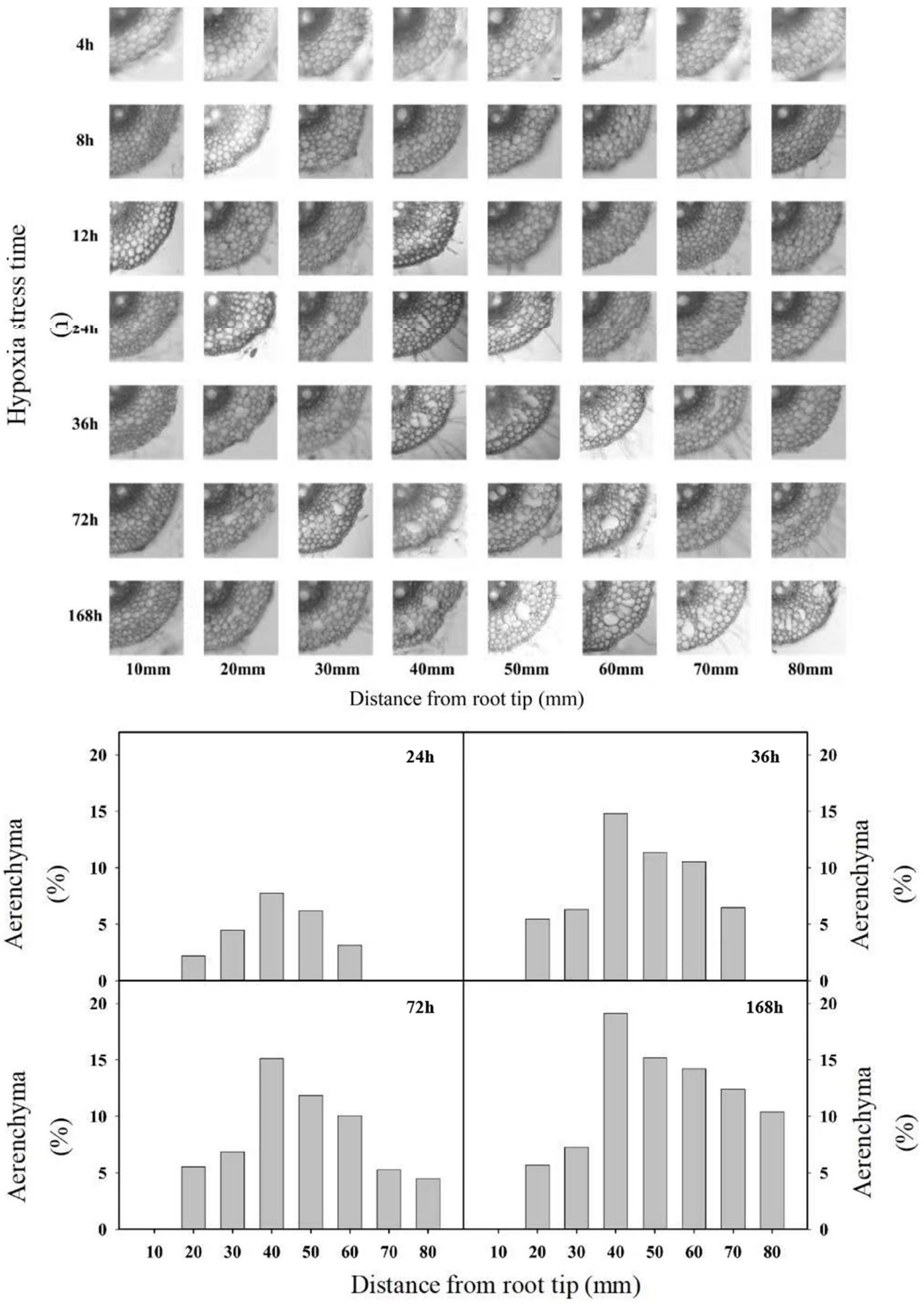

Figure S3. Effects of hypoxia stress on the formation of aerenchyma in wheat secondary roots

Supplementary Tables

Table S2. PCR primers used for semi-quantitative and quantitative RT-PCR

Gene	Forward primer 5'-3'	Reverse primer 5'-3'	Citation
TaAINV	AACGTCACAAGGCTCGTCGT	ATGTAGGCCTGATTGTAGGAGGAGT	[1]
TaNIN	CACTGGAGCGTAAGAGGTCATT	CCACACTATCAAAGCCGTCAT	[1]
TaSuS	CCGACAAGGAGAAGTATG	CGAGTTCACTAACATTCAC	[1]
TaPDC1	AGGGTCTCTGCTGCCAAC	TATTTGATGGCAACGTGCTG	[2]
TaPDC2	GAGCTTCTTGAGTGGGGTTCT	ATGTGTCACTGGGGATTTGG	[2]
TaADH1	CCCAATGTCGTGGAGATGTA	CTAGTTCTCCATGCGGATGAT	[2]
TaADH2	GGAGCTGGACGTGGAGAA	GAACGCCGTGTTGATCTG	[2]
TaACS2	AGCACCAGCAGCAGAAGG	GTGGACGAGCGGAGACTG	[2]
Actin	GCTCTCCAACAACATTGCCAA	GCTTCTGCCTGTCACATACGC	[3]

Note: *TaAINV*: Acid Invertase gene; *TaNIN*: Alkaline/neutral invertase gene; *TaSuS*: Sucrose synthase gene; *TaPDC1*, *TaPDC2*: Pyruvate decarboxylase gene; *TaADH1*, *TaADH2*: Alcohol dehydrogenase gene; *TaACS2*: 1-aminocyclopropane-1-carboxylate (ACC) synthase gene.

References:

1. Liu, L.; Cang, J.; Yu, J.; Wang, X.; Huang, R.; Wang, J.; Lu, B. Effects of exogenous abscisic acid on carbohydrate metabolism and the expression levels of correlative key enzymes in winter wheat under low temperature. Biosci Biotechnol Biochem 2013, 77, 516-525, doi:10.1271/bbb.120752.
2. Yamauchi, T.; Watanabe, K.; Fukazawa, A.; Mori, H.; Abe, F.; Kawaguchi, K.; Oyanagi, A.; Nakazono, M. Ethylene and reactive oxygen species are involved in root aerenchyma formation and adaptation of wheat seedlings to oxygen-deficient conditions. J Exp Bot 2014, 65, 261-273, doi:10.1093/jxb/ert371.
3. Wang, X.; Zhang, X.; Chen, J.; Wang, X.; Cai, J.; Zhou, Q.; Dai, T.; Cao, W.; Jiang, D. Parental drought-priming enhances tolerance to post-anthesis drought in offspring of wheat. Front Plant Sci 2018, 9, 261, doi:10.3389/fpls.2018.00261.
